# Identity, community and care in online accounts of hereditary colorectal cancer syndrome

**DOI:** 10.1080/14636778.2018.1469974

**Published:** 2018-05-02

**Authors:** Emily Ross, Tineke Broer, Anne Kerr, Sarah Cunningham-Burley

**Affiliations:** a Centre for Population Health Sciences, Usher Institute of Population Health Sciences and Informatics, Medical School, University of Edinburgh, Edinburgh, UK; b School of Sociology and Social Policy, University of Leeds, Leeds, UK

**Keywords:** colorectal cancer, temporality, hereditary cancer syndrome, genetic risk, internet research

## Abstract

Sociological literature has explored how shifts in the point at which individuals may be designated as diseased impact upon experiences of ill health. Research has shown that experiences of being genetically “at risk” are shaped by and shape familial relations, coping strategies, and new forms of biosociality. Less is known about how living with genetic risk is negotiated in the everyday and over time, and the wider forms of identity, communities and care this involves. This article explores these arrangements drawing on online bloggers’ accounts of Familial Adenomatous Polyposis (FAP). We show how accounts of genetic risk co-exist with more palpable experiences of FAP in everyday life, notably the consequences of prophylactic surgeries. We consider how the act of blogging represents but also constitutes everyday experiences of hereditary cancer syndrome as simultaneously ordinary and exceptional, and reflect on the implications of our analysis for understanding experiences of genetic cancer risk.

## Introduction

The expansion of screening programs to identify early signs of disease (Armstrong 1995), characteristic of the contemporary era of “biomedicalisation” (Clarke *et al*. 2003), has seen the emergence of categories of “pre-disease” (Kreiner and Hunt 2014). This has entailed the medical management of disease risk e.g. for high blood cholesterol (Gillespie 2012), diabetes and hypertension (Kreiner and Hunt 2014), and cancer (Fosket 2010). Being “at risk” of disease in the absence of symptoms significantly transforms individuals’ medical trajectories, but also their identities and social lives (Aronowitz 2009; Gillespie 2015). Making sense of quantifications of susceptibility to disease and biomedical classifications involves grappling with considerable uncertainties about risks, symptoms and evasive actions (Gillespie 2012), creating new kinds of identities as “pre-symptomatic persons” (Konrad 2005), or “perpetual patients” (Finkler 2000).

Authors have explored how these at-risk identities may play out within familial settings, with interpretation and disclosure mediated by disease histories, familial roles and personal biographies (Arribas-Ayllon, Sarangi, and Clarke 2008). The relevance of hereditary risk in everyday life, including reflections on its possible inheritance, are tied to shifting and complex family relations, and (social and physical) proximities to affected family members (Cox and McKellin 1999; Hallowell et al. 2006). The ability to diagnose individuals as genetically at-risk is also implicated in the formation of wider social relationships. Anthropologist Rabinow (1992), commenting on the development of genetics and molecular biology, describes that technoscientific modes of reflecting on one’s self, in terms of individual biology, can be collectivizing. For example, being designated as at (genetic) risk of disease allows for novel relationships, not limited to those sharing biological characteristics e.g. a specific mutation, but including with affected family members (see Gibbon 2008). In some cases these entail a mutual goal of developing new mechanisms to act upon risk, through activism and fundraising (Rabeharisoa 2006).

In what follows we explore personal experiences of a hereditary colorectal cancer syndrome as documented within online spaces, and the interplay of these with accounts written by others. Hereditary cancer syndromes, including Hereditary Breast and Ovarian Cancer (HBOC) and Lynch Syndrome, predispose individuals to particular, and sometimes several, cancer types. In many cases, the genetic mutations which cause these syndromes are identified in the absence of physical symptoms: initial identification of the condition is often predicated upon a strong family history of cancer(s), particularly were these have been diagnosed at a young age (Sijmons 2010). A designation of hereditary cancer risk prompts an array of emotions and sense-making activities amongst those affected. Where heightened of risks of malignancies associated with inherited cancer syndromes may be presented within clinical environments as “sanitised”, statistical probabilities, in practice these are deeply emotive, impacting upon corporeality and sense of self (Hallowell 2006; Howard et al. 2011). Individuals have been shown to make sense of these risks by drawing on personal biography, and may use relatives’ experiences of cancer as “reference points” (d’Agincourt-Canning 2005; Hesse-Biber 2014b), rather than relying on biomedical estimations of cancer risk (Kenen, Ardern-Jones, and Eeles 2003a). Living with a high risk of hereditary cancer may also entail an obligation to navigate familial risks (Douglas, Hamilton, and Grubs 2009; Bartuma, Nilbert, and Carlsson 2012), evoking feelings of responsibility and blame with regards future generations inheriting the condition (Mireskandari et al. 2009; Mozersky 2012). Managing risk through regular screening and/or preventative surgery (Hesse-Biber 2014a) and coping with symptoms (Fritzell et al. 2010; Hallowell et al. 2016), also takes a heavy emotional toll on those living with these inherited conditions.

In the context of the affective and embodied consequences of hereditary cancer syndromes, those with HBOC in particular have been shown to come together within on- and off-line spaces and form biosocial communities, by identifying themselves as “previvors”, a term used to capture their situation as surviving a pre-disposition to cancer (Pender 2012; Finer 2016; Dean 2016b). Formed around the “new truths” produced by molecular biology (Rabinow 1992; Navon 2011), the identification of oneself as a previvor has been described as empowering those carrying the BRCA1/2 mutation, providing them with a means of acknowledging the struggles in confronting and acting upon their genetic mutation (Hesse-Biber 2014b). However, we know from other research on living at risk of other genetic conditions such as Huntington’s disease (Etchegary 2010), or Duchenne Muscular Dystrophy (Parsons and Atkinson 1992), that individuals also adapt to living with genetic risk across the life course, including through efforts to “bracket off” risk (Etchegary 2010, 643) and minimize its impact on identity and everyday life. Risk can also be experienced as “latent”, only coming to the fore in the context of discussions of relationships and reproduction, and otherwise embedded in what Parsons and Atkinson describe as a “continuous process of definition and redefinition”, as individuals construct and negotiate everyday realities (1992, 453). This suggests that living at genetic risk of cancer might involve ordinary and mundane practices of identity-work and relationality, which are not experienced as exceptional or even burdensome when placed in their wider context of everyday life.

In this article, we explore how living with a genetic risk of cancer is negotiated by people living with Familial Adenomatous Polyposis (FAP). This is a hereditary cancer syndrome predisposing individuals to a range of cancers, predominantly colorectal; the risk of developing colorectal cancer within these individuals approaches 100% without surgery (Kalady and Church 2015). We attend to the management of genetic risk in the everyday through an analysis of blogs^1^ written by affected individuals, and reflect on exceptional and ordinary risks, identities, communities and practices of care therein. In recent research, sociologists have shown that blog authorship can provide individuals with a means of configuring their illness experience in terms of a coherent story, offering a sense of control in the context of the uncertainties of serious illness (McCosker 2008). More than this, argues Kotliar (2016), the public nature of blogs promotes dialogue with others experiencing the same health condition, allowing authors to “emerge from their seclusion and form rare communities with each other”, and not only create but collaboratively reconstruct narratives of ill health (2016, 1211). For illnesses where aetiology is uncertain, this may contribute to coping and even recovery (*ibid*). Blogs also give us insight into the fluidity of conceptualisations of illness and experiences of embodiment (Coll-Planas and Visa 2016), incorporating the dynamics of both adopting and challenging dominant approaches to risk and clinical care. Blogging as a practice may be thus considered productive of self-expression and illness subjectivities. As highlighted by McCosker and Darcy (2013, 1269), drawing on Frank (1995), through writing one’s story online, the truth of illness “is not only what *was* experienced, but equally what *becomes* experience in the telling and its reception”. Blog authorship, and the resulting texts, can thus be seen as constitutive, not only reflective, of illness experience. This resonates with Lupton’s (2017) more recent reflections on the “affective atmospheres” created by online spaces, which she argues contribute to emotional wellbeing and good health through the alleviation of isolation (2017, 7), but also by providing a space for activist voices. This includes the voices of “biological entrepreneurs” (Stage 2017), who use the internet to raise awareness and/or funds for cancer research, or support for other patients.

In what follows we pay attention to how experiences of living with FAP, and the surgeries and symptoms involved, are embedded in discussions of everyday life as documented within online blogs. We draw attention to the kinds of identity and emotional work that living with hereditary colorectal cancer risk entails. We present our findings in terms of three key practices within these blogs: constituting genetic and other kinds of identities in relation to family, diagnosis and surgery; community building with multiple others; and performing care via the maintenance of positivity, vigilance and endurance across time. Throughout we explore how exceptional risks and identities are constructed alongside other kinds of ordinary risks and identities, beyond a genetic susceptibility to cancer. We add to existing literature on genetic risk by foregrounding personal accounts of an inherited susceptibility to colorectal cancer, and its implications, over time. Exploring this through blogs enables an appreciation of how and why particular aspects of living with cancer risk are articulated and shared within online spaces, and the implications of this practice for (pre-)illness experience.

## Methods

We began our study with the aim of understanding how living with genetic cancer risk cancer was accounted for in online blogs by affected individuals. Exploratory scoping searches, using the search engine *Google,* found that many publically accessible blogs focus on the BRCA1/2 mutations, reflecting the higher public profile of HBOC amongst hereditary cancers (see Dean 2016a). Though fewer in number, initial readings of blogs addressing experiences of colorectal cancer risk revealed that these added an alternative lens to existing work on hereditary cancer syndromes. This was partly because there has been less research on the experiences of living at genetic risk of colorectal cancer, despite those with the condition constituting approximately 5%–10% of all colorectal cancer cases in the United States (Lynch and de la Chapelle 2003); a similar proportion to HBOC’s contribution to all breast cancer cases (Couzin 2003). Focusing our attention on colorectal cancer syndromes would enable us to make a unique contribution to the field, and to bring these experiences into dialogue with a more extensive body of research on HBOC. In addition to reflections on the meanings of risk for future self and family, hereditary colorectal cancer blogs also included extensive discussion of the everyday business of living with the interventions that had been performed to reduce the risk of cancer developing, usually a colectomy (after which the bowel function is restored through an ileo-anal pouch (J-pouch) or ileostomy (stoma)), and the periods of recovery involved. These interventions have life-limiting consequences, either directly or indirectly resulting from these surgeries (Fritzell et al. 2010). We therefore decided to focus our analysis on hereditary colorectal cancer blogs, which posed interesting case studies to explore cancer risk as lived, with individuals arguably rendered patients by these interventions themselves.

To identify relevant blogs to be taken forward to analysis we performed *Google* searches for the term “personal blog”, along with each of the conditions entailing a known genetic mutation(s) associated with heightened colorectal cancer risk^2^. These searches took place in January – April 2016, with ethical approval to reproduce extracts from blogs in academic research granted by the University of Edinburgh Research Ethics Committee. These searches identified twelve personal blogs. Eight were written by those living with FAP, three by those with Lynch syndrome, and one authored by an individual with Cowden syndrome. On reading these, the breath of experiences and interventions identified both within and between conditions led to a narrowing of focus for the research. The genetic mutations associated with FAP have near 100% penetrance rates, and as such, major surgeries are generally experienced by all those with the condition, and at an earlier stage in the life course. Because of the uniqueness of these experiences, only the eight blogs written by those living with FAP were taken forward into analysis, and are included in this article.

Of the eight blogs focusing on experiences of FAP, six were written by female bloggers, and two by male bloggers. Authors resided in the United States, Canada and Australia. Three were ongoing, with the authors’ most recent posts made at the time of the searches. Demonstrating the variability of author activity, the longest period spanned by a blog was over 5 years, and the shortest was 4 months. This provided the opportunity to explore accounts of genetic cancer risk and its management over time. With the majority of blogs spanning over one year, authors commonly discussed events and experiences of life beyond FAP itself. Table 1, below, provides an overview of included blogs.

**Table 1.** Blogs included in analysis.

Pseudonym and age at time of writing blog	Location	Age at confirmation of condition	Medical interventions to address colorectal cancer risk	Period covered by blog	Number of posts
Andrew, 30–40	US	15	Colectomy (removal of the colon) and ileo-anal pouch (reconstruction of the rectum using the small bowel), at age 15.	13 months (ongoing)	5
Catriona, 20–30	Australia	19	Total colectomy and ileostomy (an external pouch to collect intestinal waste), at time of writing blog (age 22).	37 months (ongoing)	72
Felicity, 20–30	US	28	A total colectomy with temporary ileostomy and ileo-anal pouch, at time of writing blog (age 29).	47 months	144
Hannah, 20–30	US	8	Total colectomy and majority of the small intestine removed. Temporary ileostomy followed by ileo-anal pouch, at age 9.	47 months (ongoing)	120
Helen, 30–40	US	8	Colectomy and temporary ileostomy, followed by ileo-anal pouch, at age 9.	10 months	25
Lucinda, 20–30	US	14	Anti-inflammatory medicine, no surgery at time of writing blog.	13 months	28
Natalie, 30–40	US	12	Subtotal colectomy, a temporary ileostomy and ileo-anal pouch at early age. Whipple (removal of part of the pancreas, gall bladder and duodenum), at time of writing blog.	66 months	82
Simon, 30–40	Canada	16	Whipple, colectomy and permanent ileostomy, at time of writing blog (age 33).	4 months	10

To facilitate qualitative data analysis, each blog was converted into a text file by copying the content, including images, into separate Microsoft Word documents. These documents were analysed to build a picture of each author’s family history, diagnosis, medical interventions and long-term impacts of the condition and interventions, with a summary account of each blogger’s experiences developed within a separate document. During a second reading, each blog was then read with attention to common events, metaphors and emotions observed among all eight blogs, and those that resonated with existing sociological literature, with these highlighted by the first author. Following these two readings of each blog in its entirety, extracts of text representing similar themes or issues were grouped together and labeled with an appropriate signifier, in themes and sub-themes; for example, “references to cancer” encompassed metaphors, as well as concepts including “inevitability of cancer” as well as “rejecting cancer narratives”. This process was repeated a second time, in discussion with co-authors, and generated a smaller set of wider key themes felt to reflect the content of the dataset as a whole. Below, the data are presented according to an analytical interest in the kinds of identities, communities and caring practices we found across the blogs. We reflect on where the management of genetic and other kinds of risks, and their consequences, featured within blog posts. We also attend to claims to both uniqueness and exceptionalism, and draw attention to the range of emotions documented within blogs, from expressions of hope and positivity, to doubt, anger and loss.

The use of online material for social research provokes novel ethical considerations and guidance, bringing to the fore issues of informed consent, anonymity and confidentiality. A deliberative, contextual approach is advised in the absence of national or international ethical guidelines for online research (Markham and Buchanan 2012, 2). Though the blogs we analyse are publically available, we decided to seek permission from authors to use their blogs in our published research because use of their blog as research data would likely be unanticipated, but also due to the sensitivity of some of the content. To request consent we approached the bloggers by email or through Twitter, asking permission to use their blogs, and to reproduce verbatim quotes in published work. Of the eight bloggers, seven were contacted – one was not reached due to the deletion of their website in the months following our analysis. Five authors responded positively, granting permission to reproduce extracts of their blog in published research. For those who were not able to or did not respond (Helen, Lucinda and Natalie), likely associated with inactivity on their blog, we have used their work in the development of the themes used in the paper, but have not reproduced verbatim quotes. We use pseudonyms to identify authors in the findings reported below, and exclude identifying information.

## Findings

### Identity stories

In this section we explore how bloggers performed their identities over time, starting with accounts of the reason for their blogging activity and how they narrated key events in the formation of their identity as a person at genetic risk of colorectal cancer, including in relation to their families, diagnosis and surgeries. We note here that although we construct a relatively cogent narrative in our analysis, these accounts were not necessarily presented in this order within blogs; instead reflections were entwined and scattered across blog posts, as identity stories were knitted together over time. In other words, the exceptionalism and everyday involved in these identity stories were co-produced.

All bloggers detailed reasons for starting their blog. This was not necessarily the moment of genetic diagnosis, which might be seen as a key point at which these individuals were marked out as being at exceptional risk of colorectal cancer. Instead, the catalyst was more often the life-changing surgery that ensued, in many cases several years after diagnosis. For example, Catriona, Simon and Natalie, who had tested positively for FAP several years previously, began their blog following the clinical identification of pre-cancerous polyps, and the (perceived) need for surgery to prevent the development of colorectal cancer. All documented their surgical decision-making, build-up and recovery from surgery, in a bid to support others in a similar situation, or in Natalie’s case, keep friends and family updated on her progress.

For others their blog was not prompted by a particular catalyst, but by a felt need to offer and receive support and raise awareness. For example, Felicity’s FAP was a result of a spontaneous, *de novo* mutation, and for her a diagnosis of FAP and subsequent colectomy occurred within weeks of each other. She documented her surgery and recovery online to “tell her story”, and appreciated reader comments on individual posts for letting her know she was being listened to, which made her “feel good” (August 2008). Having experienced surgery during childhood, Andrew and Helen described their reasons for commencing their blogs in terms of raising awareness of the condition. Andrew cited Felicity’s blog, expressing that he wanted to “help” others, in the same way that similar blogs, including Felicity’s which he mentioned by name, had helped him (November 2014). For Lucinda, blogging took place outwith the context of surgery, which she had not (yet) experienced. Lucinda described how she started writing her blog after being inspired by sharing her experiences in a class presentation. Blogging was portrayed as a way to meet others with FAP who she was not related to, which allowed her to appreciate that everyone’s experiences of FAP are different, and that “her disease” is unique (November 2012).

The experience of diagnosis was, however, an important point of reference within all of the blogs we reviewed, often described early on, and framed within a wider account of how FAP became a presence within their lives. Such accounts drew on vivid reflections on their childhoods, surgeries, and family histories. For Andrew, Catriona, Lucinda, Hannah and Helen, who emphasized the hereditary nature of the condition, diagnosis was described in the context of detailed memories of family cancer histories. For example, Lucinda described in her first post that cancer had always been present in her life, with her father being diagnosed with colon cancer, and subsequently FAP, when she was three years old (May 2012). In a post entitled *Identity* (April 2013), she reflected that it is because of such experiences that she felt her disease had defined her, becoming a part of her life before she knew she had it. Helen started her blog with memories of family members “dropping like flies” throughout the generations (October 2013). Hannah discussed similar reflections at the commencement of her blog, describing her “battle” with FAP as heavily shaped by familial experience of cancer and death (May 2012). These thoughts on shared familial experience of her condition re-emerged the following year, as she dedicated a post to colon cancer awareness month. She described:

FAP has ran in my family for many generations … I think of the numerous ancestors my family has lost to colon cancer and how it has touched each of my family members. *Hannah, March 2013*

These accounts situate the experience of FAP as one of shared familial risk and sorrow, marking the experience as both exceptional and endemic within the context of their own families. However, bloggers also juxtaposed this sense of sadness with positivity about the benefits that shared family experiences could bring to their efforts to manage their condition. For example, Hannah reflected positively on her mother’s shared experiences of FAP, which she felt gave her a unique understanding of her own health issues, and prepared her for her own life of ostomies, operations, and the “skills” needed to overcome these.

Bloggers also gave accounts of regrets and sense of good fortune in relation to the much remarked upon 50/50 risk of inheriting FAP (an autosomal dominant condition) across their families, in discussions of what might be or what might have been for other family members. For Helen, this concerned the experience of her sister, who had attended a colonoscopy in childhood to screen for FAP at the same time as herself, but was found to have escaped the condition (November 2013). Helen described an initial feeling of jealousy towards her sister, and a sense of “why me?”. In a later post, she also described feelings of envy towards her sister whose growth in adolescence, unlike hers, had not been hindered by ongoing bouts of surgery and subsequent illnesses (November 2013). Andrew compared his experiences of FAP with those of a paternal cousin, with both diagnosed with the “exact same strain of FAP” during their teenage years, and experiencing colectomies at a similar time. Whilst he writes that years of living with FAP had been kind to him, he contrasted this with his cousin, who during the same period had undergone several more major surgeries with severely life-limiting consequences, including a feeding tube and severe weight loss (September 2015). Within these accounts Helen and Andrew worked through their feelings of confusion and frustration about how experiences of FAP could differ so much between individuals. The possibilities of passing the condition on to future generations was also discussed by several bloggers who talked about “breaking” or “ending the family cycle” (Hannah, July 2013). Where Lucinda hoped that this may be achieved through the discovery of cure for FAP, Hannah, Helen and Felicity discussed their plans to either forgo having their own children, or embarking upon prenatal genetic diagnosis and IVF to ensure the condition would not be passed on. In his final post (June 2015), Simon reflected on his plans to adopt children with his wife. Through these kinds of accounts, and with reference to the blogs of others, bloggers “collaboratively reconstructed” (Kotliar 2016) their narratives and family histories.

Another important feature of the blogs we analysed was authors’ discussion of surgery, not just as a prompt for beginning the blog, but as a constituent part of their identity stories. Bloggers described the prospect of (further) major surgeries in the absence of physical symptoms, often juxtaposing this with the experiences of other health conditions. For example, whilst documenting his preparation for a colectomy, Simon explained:

I am a healthy person now; I have no symptoms. Most people when considering a permanent ileostomy either have some form of disease such as colitis, cro[h]ns, IBD or cancer OR they have suffered a trauma such as a car accident or a gunshot wound. I fit neither of these cases and I feel great day to day. *Simon, November 2014*

Experiences of the difficulties and complications following surgery were also discussed at length. Natalie, reflecting on her experiences four years after the Whipple procedure, described how living with FAP was unusual, particularly because surgeries such as Whipple and colectomy are generally performed on those living with Crohn’s disease or pancreatic cancer. In her case she had experienced “no problems”, until her surgery caused “lots of problems”, and she described having to transition to a “new normal” (November 2011). Hannah too had reflected on this, and described the disease, and its (indirect) consequences, as “complicated”:

I don’t view my bowel issues as a problem because of my FAP but instead it’s a result of complications from surgeries … FAP sucks and is a horrible disease, but by itself it isn’t necessarily such a god awful disease. *Hannah, October 2012*

For these authors, then, their experience of the complications and life-limiting consequences of surgeries in the absence of prior ill-health presented them with difficult risk calculus to manage, before and after surgery. Identity-stories drew heavily on experiences of taking and living with the physical risks associated with major surgery and colostomy to offset prospective genetic risks of cancer. This rendered them exceptional or unusual in relation to other individuals experiencing these surgeries, but it also produced similar kinds of experiences post-surgery to be navigated together with those undergoing these procedures for other conditions. The embodied risks of surgery came to the fore as a key part of these identity-stories, as did the daily consequences of living with ileostomy, and managing associated complications such as anaemia and fatigue.

The genetic mutation associated with FAP featuring in the identity-stories of these authors was presented at times as a bounded entity that passed through the family, or as Helen described “plagued” their genes. This involved stories of exceptionalism, as in Helen’s case where she described being aware from a young age that this mutation made her different from others, and contributed to feelings of loneliness as a child (November 2013). Identity stories also involved navigation of being different within families: being exceptionally lucky or unlucky as compared with others. In other respects families were marked out as exceptional, as captured in Catriona’s remarks that for her and her family, “deep down in our DNA we literally have a ticking time bomb within us” (December 2014). The prospects of having surgery in the absence of ailments also marked people with FAP as different from others facing or experiencing similar surgeries, but they also shared the long-term consequences of these interventions. These stories of exceptionalism are, thus, entwined with stories emphasizing similarities, or the search for commonality beyond the specifics of genetic risk; the desire to share experiences and navigate embodied physical risks and decision-making around reproduction or surgery, to educate, to offer or receive support, and to form links with people experiencing other kinds of surgeries or chronic illness. We now go on to explore in more depth this community building and care-work.

### Community building

Online blogs functioned as spaces for building connections with multiple audiences, including family, friends, and individuals with similar experiences. This included people affected by FAP, and those undergoing similar surgeries or chronic or rare diseases more generally. Bloggers sought connections with others experiencing FAP, including via sharing accounts of the poor understanding of their condition amongst medical professionals and within their social circles. This sense of a need to offer and receive support was described by Natalie as a result of having to “be her own advocate”, when encountering doctors who lacked awareness that she could be at risk of colon cancer at such a young age (November 2012). Throughout his blog Simon also described blogging as a way of supporting others with similar experiences, whom Natalie described as fellow “gutless” brothers and sisters (October 2012).

Authors drew heavily on their experiences of medical procedures and complications as a way of providing support to others, but also to raise awareness of their condition. The impacts of surgeries were foregrounded within blogs, with the physical consequences described in detail. These included anaemia, experienced by Hannah, Natalie and Felicity, digestive issues, and at times physical and/or emotional discomfort for those who lived with an ileostomy bag. Blogs were generally oriented to providing advice on how to manage post-surgery, but others were more overt in encouraging people to proceed with medical interventions: for example, a key aim for Helen was to encourage her readers, not only those with FAP, to embark on a colonoscopy. She acknowledged this procedure was daunting, but aimed to de-mystify colonoscopies through her writing (March 2014).

The giving of advice included detailed discussion of how to deal with the ongoing physical impacts of these interventions and surgeries. For Andrew, Hannah, Natalie and Felicity, who lived with a J-Pouch, an example of this was the need to plan their daily activities around access to restrooms:

Planning for an activity away from home and specifically in a location you’re not sure about the access to restrooms, is tiring and difficult at times … For those living with bowel and bladder disorders, this is a huge concern. I feel as though my life revolves [around] a toilet. What a disgusting thing for my life to revolve around. *Hannah, July 2012*

Coping with the stigma of a stoma at a young age was a frequent source of discussion for those living with one. Catriona’s blog was motivated by a felt need for support on living with a stoma. She explained:

I knew how hard it was for me (who was 22 when I had my surgery to remove my entire large bowel) to find information and support that was aimed at a younger person as everything I found was more suited to the older population or geriatric care respectively. So I realised that if I was struggling that there was bound to be others out there struggling as well. *October 2014*

In posts such as “5 Ways To Make Your Stoma Bags More Stickier” (October 2014), and “5 quick fashion tips when you have a stoma” (April 2016), Catriona provided practical advice to other young adults living with this intervention. In common with Felicity, Natalie and Simon, Catriona also posted accounts of her surgery and at times photos, to inform readers about their procedures and recovery. Importantly, the audiences that these posts were targeted at, and thus communities of imagined others with shared experiences, traveled beyond FAP. With the effects of the interventions designed to manage cancer risk being the most common focus of posts, blogs were addressed to those living with a wide range of conditions. For example, Andrew’s blog contained links to a wide variety of forums including those of individuals living with ulcerative colitis and Crohn’s disease, which also often entail a colectomy. The community building thus extended beyond genetic risk of cancer to a wider, shared experience of colon surgeries, chronic conditions and indeed other rare diseases.

Beyond colorectal conditions, we also found that some bloggers identified with a wider network of “chronic illness bloggers”, connecting with not only those sharing their syndrome or post-surgery experiences of stoma, but those sharing experiences of frequent hospitalization, and a lack of understanding from the “non-sick”. Hannah called this a “chronic illness camaraderie”:

For those of us with a rare disease or other chronic illness, it doesn’t take much to relate to one another regardless of the diagnosis. Our commonalities create an instant bond, an instant understanding of another’s life with chronic illness. *Hannah, January 2016*

Lucinda also identified with the wider rare disease community, and regularly fundraised for a charity advocating for those with rare and genetic diseases. For Lucinda, finding support from this community was an unexpected but welcome consequence of beginning her blog, which had attracted many readers and begun to lead to links “offline”, which she then described on her blog.

This suggests that, for bloggers with a heightened risk of colorectal cancer attributable to FAP, their exceptional genetic risk was not a dominant or consistent focus of their writing, but a theme which jostled with accounts of shared experiences of medical interventions and their embodied consequences, together with advocacy for wider societal understandings and acceptance. Hereditary cancer risk was not a constant presence, and accounts of experiences were adopted and adapted to share with a wide range of audiences with similar and sometimes quite diverse experiences. The sharing of experiences was described by all authors as a key reason for beginning and continuing with their blog; as a way to help others overcome hurdles that they themselves had been through. Through these practices, blogging built communities. The performance of self-care and care for others was a key feature of this, and is discussed further below.

### Care as maintenance

Care encompassed a range of emotional and embodied practices to offer support, sympathy, protection and relief from pain and discomfort. Typically performed in institutionalized as well as in domestic, private settings, care can also be found in virtual worlds, enacted through texts and images, and crucially feelings and experiences shared amongst and between individuals. Importantly, care also unfolds over time, as an “arduous temporal practice” oriented towards the maintenance of selves and others (Baraitser 2017, 29). In this section we explore how some of these practices unfolded in the blogs we analysed, looking more closely at articulations of positivity, vigilance and endurance in the face of genetic cancer risk.

The blogs we reviewed placed considerable importance on living well over time, by successfully navigating risks in all shapes and sizes. For the most part, though acknowledged as difficult to overcome, and requiring the support of others (including anonymous bloggers and readers), the impacts of surgeries were presented as an acceptable trade-off for the reduced risk of colorectal cancer they offered. This was evident in Natalie’s blog, who exclaimed in November 2012 that although living without digestive organs hadn’t been easy, she feels “blessed” to have avoided cancer. Expressing similar sentiments, Felicity explained:

I am constantly reminded of how lucky I am. In the whole scheme of things, I’m not sick. Yeah I lost an organ … but I avoided chemo right?! … The crap I deal with now is really just small potholes when compared to the craters that others have to deal with. *Felicity, November 2008*

Bloggers were at times positive about knowledge of their heightened cancer risk, describing that this knowledge had provided them with an opportunity to act upon risk, by undergoing screening to address (inevitable) tumors at an “early” stage of development:

Yea you’re guaranteed to develop cancer at some point, but caught early enough is easily treated and likely will only require surgery as treatment option. *Hannah, October 2012*

Many of the blogs included posts about strength and positivity regarding their surgeries, with bloggers maintaining a hopeful perspective on their condition. All bloggers at one time or another reflected on the condition as having improved their lives, by encouraging them to “live each day to its fullest” (Andrew, May 2015) or teaching them that nothing is impossible (Lucinda, December 2012). The sharing of these positive experiences was a means by which some bloggers felt they could care for others living with the same, or another, health condition. For example, Simon described:

Reading other people’s blogs such as [Felicity’s blog] who have FAP and/or had a colectomy has been helpful. Along these lines I thought my sharing my experience through a blog could be helpful to others in a similar situation and could also help me through the experience as well. *Simon, November 2014*

In her first blog post, Lucinda hoped that sharing her story would change another person’s life, by helping another through a difficult time (May 2012). However, whilst reading blogs across time, we also found that maintaining a positive approach was a process of struggle, of confronting reappearing threats and insecurities about cancer. For example, having endured prophylactic surgery, and at times reflecting positively on his outlook, cancer risk remained present for Andrew:

[I have known] that my future would always be in question because of this disease. *Andrew, May 2015*

Simon similarly discussed that:

I have also learned that living with FAP can mean living with a lot of what-iffs … These what-iffs can escalate quickly to the point as if it feels like death is knocking on my door. *Simon, November 2014*

For some bloggers, these anxieties were raised at the time of anniversaries or specific clinical appointments. Lucinda described having nightmares about being diagnosed with cancer, occurring most frequently as she prepared to undergo yearly screening (April 2013). Because of similar anxieties, Helen reflected on whether not knowing about their condition would be preferable, citing the refrain “ignorance is bliss” (October 2013).

These re-emerging fears and concerns prompted bloggers to be vigilant for emergent signs of cancer in the knowledge that further risk-reducing surgeries were an ever-present possibility. These narratives performed an ongoing form of maintenance, combining reassurance and vigilance in the face of threats and risks, or in the words of Natalie, a “never-ending battle”, and a “journey with no end” (January 2013). Lucinda too used the term “rollercoaster” (August 2012) to describe the ups and downs of FAP, which shifted between attaining a sense of normalcy, and raised anxiety prior to her screenings.

Blogs were more explicitly presented as a tool of self-care when the act of blogging was performed for the purposes of “offloading” particular opinions or negative experiences. Three blog authors, Natalie, Felicity and Hannah, wrote individual blog posts which they described as occasions to “rant” or “vent” about their situation. Natalie used her blog to record her thoughts when having what she described as “one of those days”, which included reflections on the consequences of her surgery for her relationships with her children, and negative encounters with those clinicians who did not take her lived experience and knowledge of her condition seriously (November 2011). She called blogging a form of “de-stressing” therapy. Indeed, for many authors blogging was a significant aspect of living with FAP and life post-surgery, and in the (emotional) management of their condition:

Judging from the story lines though of the 5 or 6 showings of [my] subconscious per night I think this is where the heavy lifting is being done on my fears, anxieties and feelings of hurt/loss associated with my medical journey … I am going to see if doing some of the heavy lifting during the day might result in some relief. Along these lines I am going to continue with regular physical activity … journal writing each day AND last doing some more blogging. *Simon, February 2015*

Here blogs are being used as a form of self-care in the face of negative experiences, fears and anxieties. This is another form of maintenance, of sustaining and preserving a positive outlook and of endurance of complex emotions and ambivalence. Once more, blog posts combined an appreciation of being exceptional with references to shared and ordinary practices of living well. These included the management of emotions, with blogs functioning across time as expressions of positivity, help and resolve, interspersed with the articulation and management of disgruntlement and despondency. Through these practices blogs perform care as a combination of the exceptional and ordinary maintenance of bodies, selves and futures.

## Discussion and conclusion

In this article, we have explored the blogs of eight individuals living with a high risk of colorectal cancer to understand how identities, communities and practices of care operate therein. We have traced how and when the exceptional genetic risk of FAP is articulated and managed across time, and in relation to other kinds of risks such as those associated with preventative surgeries, and with respect to other individuals with varying health conditions.

Authors’ posts also gave insight into how participants conceived of their condition as in some way inscribed in their identity, through reflection on the gene mutation itself, or through memories of familial encounters with surgeries and cancer. In so doing they navigated the terrain of responsibility, guilt and loss redolent of other genetic predispositions to cancer (cf. Hallowell 2006), and the active subjecthood with which it is associated (Rose 2007). However, the bloggers considered here also engaged with a plethora of other concerns and emotions beyond the family or the gene when maintaining a positive and vigilant attitude to their health, including the articulation of doubts, fears and anger as part of their wider efforts to endure their condition, both now and in the future.

Rabinow (1992), writing about the molecularisation of medicine, prophesised that new social formations organized around molecular information, which he terms “biosocialities”, would increasingly shape the organization and practice of medicine. Indeed, this has been reported in social scientific research with those living with hereditary breast and ovarian cancer, which has described forming communities of “previvors” drawn together by virtue of shared experiences of living with the BRCA gene mutation (Pender 2012; Dean 2016b). However, in the blogs we analysed the project of community building went beyond advocacy and support for people who had experience of the specific condition of FAP, and traveled more widely to readers experiencing similar everyday impacts of surgeries as a result of conditions including Crohn’s disease and ulcerative colitis, via blogs addressing sensitive and stigmatized issues such as living with a stoma (see also Ramirez et al. 2014). We found that, although the exceptionalism of genetic risk was discussed and explored across the blogs, particularly in relation to familial relations and reasons for surgery, it was situated within a broader set of accounts of other risks and their management that were not particular to people with this condition, for example in relation to living with the aftermath of surgery, or as a person with a rare or chronic disease. The blogs we analysed were a means through which authors navigated their identities in relation to a broad set of illness experiences: which were not always framed in terms of their genetic status. Reflecting existing literature, our analysis also shows that bloggers’ accounts of experiencing hereditary cancer risk changed over time. Their appreciation of risk and the proximity of cancer waxed and waned (Etchegary 2010), with a sense of genetic risk heightening at particular points, for example in relation to yearly screening. This reflects the ambiguities of living with chronic risk reported for other conditions (Kenen, Ardern-Jones, and Eeles 2003b).

Through forming social connections with others sharing their condition, blogging contributed to a community of support, but also a means by which authors made sense of their FAP and its particularities. As described by Lucinda, hearing from those who shared her condition but were not family members helped her to reflect on the meaning of her condition in new ways, constructing it as uniquely “hers”. This combined effort to cultivate support seeking which went beyond the rarity of FAP, through shared experience alongside expressions of exceptionalism and uniqueness, seemed to allow for an acknowledgement that everyone is “different” in how they experience ill health (Mazanderani, Locock, and Powell 2012) but nonetheless encounter common concerns and experiences.

This analysis points to the multiplicities of the “affective atmospheres” (Lupton 2017) of online illness environments, even in blogs pertaining to relatively rare and circumscribed condition such as FAP. This includes emotional wellbeing, support for others and practices of maintenance and endurance, as well as the navigation of exceptional and ordinary risks and responsibilities for health. We have gained insight into the long term consequences of living with hereditary cancer syndrome, and the ways in which genetic risks jostle with everyday, ordinary risks in their expression and management, with bloggers defying fixed categories of action such as “community-building”, “support seeking” or “biological entrepreneurship” (Stage 2017). This work enriches social scientific explorations of genetic risk, particularly with regards the recognition that internet use is enmeshed with contemporary experiences of health and illness (Nettleton and Burrows 2003). Indeed, we have shown that online accounts of living with hereditary cancer syndromes may be considered constitutive of personal experiences and understandings of genetic cancer risk (McCosker and Darcy 2013), facilitating the articulation of difficult and emotional histories of cancer, catharsis, and identification with (imagined) others.

## Notes

1 Online weblogs, or “blogs”, are a form of online communication particularly amenable to self-expression. Blogs are ongoing online accounts authored by a single individual, capturing user reflections on events, opinions and personal experiences. They usually comprise of dated entries, and within posts a user can include photos and links to webpages, and comments can often be left by readers (Snee 2010).

2 Familial Adenomatous polyposis (FAP), MYH-associated polyposis (MAP), Juvenile polyposis syndrome (JPS), Peutz-Jeghers polyposis (PJP), Cowden syndrome, and Lynch syndrome (Kalady and Church 2015, 113). 

## References

[CIT0001] Armstrong D. 1995 “The Rise of Surveillance Medicine.” 17 (3): 393–404. doi: 10.1111/1467-9566.ep10933329

[CIT0002] Aronowitz R. A. 2009 “The Converged Experience of Risk and Disease.” 87 (2): 417–442. doi: 10.1111/j.1468-0009.2009.00563.x PMC2728027 19523124

[CIT0003] Arribas-Ayllon M., Sarangi S., and Clarke A. 2008 “Managing Self-Responsibility Through Other-Oriented Blame: Family Accounts of Genetic Testing.” 66 (7): 1521–1532. doi: 10.1016/j.socscimed.2007.12.022 18222583

[CIT0004] Baraitser L. 2017 . London: Bloomsbury.

[CIT0005] Bartuma K., Nilbert M., and Carlsson C. 2012 “Family Perspectives in Lynch Syndrome Becoming a Family at Risk, Patterns of Communication and Influence on Relations.” 10 (1): 1–6. doi: 10.1186/1897-4287-10-6 PMC3418549 22632157

[CIT0006] Clarke A. E., Shim J. K., Mamo L., Fosket J. R., and Fishman J. R. 2003 “Biomedicalization: Technoscientific Transformations of Health, Illness, and U.S. Biomedicine.” 68 (2): 161–194. doi: 10.2307/1519765

[CIT0007] Coll-Planas G., and Visa M. 2016 “The Wounded Blogger: Analysis of Narratives by Women With Breast Cancer.” 38 (6): 884–898. doi: 10.1111/1467-9566.12405 26893129

[CIT0008] Couzin J. 2003 “The Twists and Turns in BRCA’s Path.” 302 (5645): 591–593. doi: 10.1126/science.302.5645.591 14576418

[CIT0009] Cox S. M., and McKellin W. 1999 “‘There’s This Thing in Our Family’: Predictive Testing and the Construction of Risk for Huntington Disease.” 21 (5): 622–646. doi: 10.1111/1467-9566.00176

[CIT0010] d’Agincourt-Canning L. 2005 “The Effect of Experiential Knowledge on Construction of Risk Perception in Hereditary Breast/Ovarian Cancer.” 14 (1): 55–69. doi: 10.1007/s10897-005-1500-0 15789156

[CIT0011] Dean M. 2016a “Celebrity Health Announcements and Online Health Information Seeking: An Analysis of Angelina Jolie’s Preventative Health Decision.” 31 (6): 752–761. doi: 10.1080/10410236.2014.995866 26574936

[CIT0012] Dean M. 2016b ““It’s Not If I Get Cancer, It’s When I Get Cancer”: BRCA-Positive Patients’ (Un)Certain Health Experiences Regarding Hereditary Breast and Ovarian Cancer Risk.” 163: 21–27. doi: 10.1016/j.socscimed.2016.06.039 27376595

[CIT0013] Douglas H. A., Hamilton R. J., and Grubs R. E. 2009 “The Effect of BRCA Gene Testing on Family Relationships: A Thematic Analysis of Qualitative Interviews.” 18 (5): 418–435. doi: 10.1007/s10897-009-9232-1 19479365

[CIT0014] Etchegary H. 2010 “‘I Put it on the Back Burner Most Days’: Living With Chronic Risk.” 15 (6): 633–649. doi: 10.1177/1363459310364162 21177715

[CIT0015] Finer B. S. 2016 “The Rhetoric of Previving: Blogging the Breast Cancer Gene.” 35 (2): 176–188. doi: 10.1080/07350198.2016.1142855

[CIT0016] Finkler K. 2000 . Philadelphia, PA: University of Pennsylvania Press.

[CIT0017] Fosket J. R. 2010 “Breast Cancer Risk as Disease: Biomedicalising Risk.” In , edited by Clarke A. E., Mamo L., Fosket J. R., Fishman J. R., and Shim J., 331–352. London: Duke University Press.

[CIT0018] Frank A. 1995 . Chicago: University of Chicago Press.

[CIT0019] Fritzell K. R., Persson C., Bjork J., Hultcrantz R., and Wettergren L. 2010 “Patients’ Views of Surgery and Surveillance for Familial Adenomatous Polyposis.” 33 (2): E17–E23. doi: 10.1097/NCC.0b013e3181bb0cf1 20142744

[CIT0020] Gibbon S. 2008 “Charity, Breast Cancer Activism and the Iconic Figure of the BRCA Carrier.” In , edited by Gibbon S. and Novas C., 19–37. London: Routledge.

[CIT0021] Gillespie C. 2012 “The Experience of Risk as ‘Measured Vulnerability’: Health Screening and Lay Uses of Numerical Risk.” 34 (2): 194–207. doi: 10.1111/j.1467-9566.2011.01381.x 21848989

[CIT0022] Gillespie C. 2015 “The Risk Experience: The Social Effects of Health Screening and the Emergence of a Proto-Illness.” 37 (7): 973–987. doi: 10.1111/1467-9566.12257 25912148

[CIT0023] Hallowell N. 2006 “Varieties of Suffering: Living With the Risk of Ovarian Cancer.” 8 (1): 9–26. doi: 10.1080/13698570500532322

[CIT0024] Hallowell N., Arden-Jones A., Eeles R., Foster C., Lucassen A., Moynihan C., and Watson M. 2006 “Guilt, Blame and Responsibility: Men’s Understanding of Their Role in the Transmission of BRCA1/2 Mutations Within Their Family.” 28 (7): 969–988. doi: 10.1111/j.1467-9566.2006.522_2.x 17163862

[CIT0025] Hallowell N., Badger S., Richardson S., Caldas C., Hardwick R. H., Fitzgerald R. C., and Lawton J. 2016 “An Investigation of the Factors Effecting High-Risk Individuals’ Decision-Making About Prophylactic Total Gastrectomy and Surveillance for Hereditary Diffuse Gastric Cancer (HDGC).” 15 (4): 1–12. doi: 10.1007/s10689-016-9910-8 PMC5935221 27256430

[CIT0026] Hesse-Biber S. 2014a “The Genetic Testing Experience of BRCA-Positive Women: Deciding Between Surveillance and Surgery.” 24 (6): 773–789. doi: 10.1177/1049732314529666 24747286

[CIT0027] Hesse-Biber S. N. 2014b . Ann Arbor: University of Michigan Press.

[CIT0028] Howard A. F., Balneaves L. G., Bottorff J. L., and Rodney P. 2011 “Preserving the Self: The Process of Decision Making About Hereditary Breast Cancer and Ovarian Cancer Risk Reduction.” 21 (4): 502–519. doi: 10.1177/1049732310387798 PMC4880460 20980697

[CIT0029] Kalady M. F., and Church J. M. 2015 “Prophylactic Colectomy: Rationale, Indications, and Approach.” 111 (1): 112–117. doi: 10.1002/jso.23820 25418116

[CIT0030] Kenen R., Ardern-Jones A., and Eeles R. 2003a “Family Stories and the Use of Heuristics: Women from Suspected Hereditary Breast and Ovarian Cancer (HBOC) Families.” 25 (7): 838–865. doi: 10.1046/j.1467-9566.2003.00372.x 19774749

[CIT0031] Kenen R., Ardern-Jones A., and Eeles R. 2003b “Living With Chronic Risk: Healthy Women With a Family History of Breast/Ovarian Cancer.” 5 (3): 315–331. doi: 10.1080/13698570310001607003

[CIT0032] Konrad M. 2005 . Cambridge: Cambridge University Press.

[CIT0033] Kotliar D. M. 2016 “Depression Narratives in Blogs: A Collaborative Quest for Coherence.” 26 (9): 1203–1215. doi: 10.1177/1049732315612715 26531881

[CIT0034] Kreiner M. J., and Hunt L. M. 2014 “The Pursuit of Preventive Care for Chronic Illness: Turning Healthy People Into Chronic Patients.” 36 (6): 870–884. doi: 10.1111/1467-9566.12115 PMC4074448 24372285

[CIT0035] Lupton D. 2017 “How Does Digital Health Feel? Towards Research on the Affective Atmospheres of Digital Health.” 3: 1–11. 10.1177/2055207617701276 PMC6001181 29942587

[CIT0036] Lynch H. T., and de la Chapelle A. 2003 “Hereditary Colorectal Cancer.” 348 (10): 919–932. doi: 10.1056/NEJMra012242 12621137

[CIT0037] Markham A., and Buchanan E. 2012 [Online] Chicago: Association of Internet Researchers, Research Ethics Working Committee Accessed December 13, 2017. http://aoir.org/ethics/.

[CIT1000] Mazanderani F., Locock L., and Powell J. 2012 “Being Differently the Same: The Mediation of Identity Tensions in the Sharing of Illness Experiences.” 74 (4): 546–553. doi: 10.1016/j.socscimed.2011.10.036 22227237

[CIT0038] McCosker A. 2008 “Blogging Illness: Recovering in Public.” [Online] 11 http://journal.media-culture.org.au/index.php/mcjournal/article/view/104.

[CIT0039] McCosker A., and Darcy R. 2013 “Living With Cancer.” 16 (8): 1266–1285. doi: 10.1080/1369118X.2012.758303

[CIT0040] Mireskandari S., Sangster J., Meiser B., Thewes B., Groombridge C., Spigelman A., and Andrews L. 2009 “Psychosocial Impact of Familial Adenomatous Polyposis on Young Adults: A Qualitative Study.” 18 (5): 409–417. doi: 10.1007/s10897-009-9231-2 19479366

[CIT0041] Mozersky J. 2012 “Who’s to Blame? Accounts of Genetic Responsibility and Blame Among Ashkenazi Jewish Women at Risk of BRCA Breast Cancer.” 34 (5): 776–790. doi: 10.1111/j.1467-9566.2011.01427.x 22257279

[CIT0042] Navon D. 2011 “Genomic Designation: How Genetics Can Delineate New, Phenotypically Diffuse Medical Categories.” 41 (2): 203–226. doi: 10.1177/0306312710391923 21998922

[CIT0043] Nettleton S., and Burrows R. 2003 “E-Scaped Medicine? Information, Reflexivity and Health.” 23 (2): 165–185. doi: 10.1177/0261018303023002003

[CIT0044] Parsons E., and Atkinson P. 1992 “Lay Constructions of Genetic Risk.” 14 (4): 437–455. doi: 10.1111/1467-9566.ep10493083

[CIT0045] Pender K. 2012 “Genetic Subjectivity in Situ: A Rhetorical Reading of Genetic Determinism and Genetic Opportunity in the Biosocial Community of FORCE.” 15 (2): 319–349.

[CIT0046] Rabeharisoa V. 2006 “From Representation to Mediation: The Shaping of Collective Mobilization on Muscular Dystrophy in France.” 62 (3): 564–576. doi: 10.1016/j.socscimed.2005.06.036 16051407

[CIT0047] Rabinow P. 1992 “Artificiality and Enlightenment: From Sociobiology to Biosociality.” In , edited by Crary J. and Kwinter S., 234–252. New York: Zone.

[CIT0048] Ramirez M., Altschuler A., McMullen C., Grant M., Hornbrook M., and Krouse R. 2014 ““I Didn’t Feel Like I Was a Person Anymore”: Realigning Full Adult Personhood After Ostomy Surgery.” 28 (2): 242–259. doi: 10.1111/maq.12095 PMC5023005 24782269

[CIT1001] Rose N. S. 2007 The Politics of Life Itself: Biomedicine, Power, and Subjectivity in the Twenty-first Century. Princeton, NJ: Princeton University Press.

[CIT0049] Sijmons R. 2010 “Identifying Patients with Familial Cancer Syndromes.” In , edited by Riegert-Johnson D., Boardman L., and Hefferon T., 1–11. Bethesda, MD: National Center for Biotechnology Information. 21249759

[CIT0050] Snee H. 2010 “Using Blog Analysis.” *Realities Toolkit* [Online]. Accessed July 29, 2016. http://hummedia.manchester.ac.uk/schools/soss/morgancentre/toolkits/10-toolkit-blog-analysis.pdf.

[CIT0051] Stage C. 2017 . Cham: Springer International Publishing.

